# ‘Care and Prevent’: rationale for investigating skin and soft tissue infections and AA amyloidosis among people who inject drugs in London

**DOI:** 10.1186/s12954-018-0233-y

**Published:** 2018-05-08

**Authors:** M. Harris, R. Brathwaite, Catherine R. McGowan, D. Ciccarone, G. Gilchrist, M. McCusker, K. O’Brien, J. Dunn, J. Scott, V. Hope

**Affiliations:** 1 Department of Public Health, Environments, and Society London, School of Hygiene & Tropical Medicine, 15-17 Tavistock Place, London, WC1H 9SH UK; 2 Family and Community Medicine, University of California San Francisco, San Franciso, CA 94143 USA; 3 Institute of Psychiatry, Psychology and Neuroscience, National Addiction Centre, King’s College London, 4 Windsor Walk, London, SE5 8BB UK; 4 Lambeth Service Users Forum, Lorraine Hewitt House, Brighton Terrace, London, SW9 8DG UK; 5 Camden Drug Services, The Margarete Centre, 108 Hampstead Road, London, NW1 2LS UK; 6 Department of Pharmacy and Pharmacology, University of Bath, Claverton Down, Bath, BA2 7AY UK; 7 Public Health Institute, Liverpool John Moores University, 79 Tithebarn Street, Liverpool, L2 2ER UK; 8 Humanitarian Public Health Technical Unit, Save the Children UK, London, UK

**Keywords:** AA amyloidosis, People who inject drugs, Kidney disease, Skin and soft tissue infections, Harm reduction, Mixed methods, Protocol

## Abstract

**Background:**

Skin and soft tissue infections (SSTIs) are a leading cause of morbidity and mortality among people who inject drugs (PWID). International data indicate up to one third of PWID have experienced an SSTI within the past month. Complications include sepsis, endocarditis and amyloid A (AA) amyloidosis. AA amyloidosis is a serious sequela of chronic SSTI among PWID. Though there is a paucity of literature reporting on AA amyloidosis among PWID, what has been published suggests there is likely a causal relationship between AA amyloidosis and injecting-related SSTI. If left untreated, AA amyloidosis can lead to renal failure; premature mortality among diagnosed PWID is high. Early intervention may reverse disease. Despite the high societal and individual burden of SSTI among PWID, empirical evidence on the barriers and facilitators to injecting-related SSTI prevention and care or the feasibility and acceptability of AA amyloidosis screening and treatment referral are limited. This study aims to fill these gaps and assess the prevalence of AA amyloidosis among PWID.

**Methods:**

Care and Prevent is a UK National Institute for Health Research-funded mixed-methods study. In five phases (P1–P5), we aim to assess the evidence for AA amyloidosis among PWID (P1); assess the feasibility of AA amyloidosis screening, diagnostic and treatment referral among PWID in London (P2); investigate the barriers and facilitators to AA amyloidosis care (P3); explore SSTI protection and risk (P4); and co-create harm reduction resources with the affected community (P5). This paper describes the conceptual framework, methodological design and proposed analysis for the mixed-methods multi-phase study.

**Results:**

We are implementing the Care and Prevent protocol in London. The systematic review component of the study has been completed and published. Care and Prevent will generate an estimate of AA amyloidosis prevalence among community recruited PWID in London, with implications for the development of screening recommendations and intervention implementation. We aim to recruit 400 PWID from drug treatment services in London, UK.

**Conclusions:**

Care and Prevent is the first study to assess screening feasibility and the prevalence of positive proteinuria, as a marker for AA amyloidosis, among PWID accessing drug treatment services. AA amyloidosis is a serious, yet under-recognised condition for which early intervention is available but not employed.

**Electronic supplementary material:**

The online version of this article (10.1186/s12954-018-0233-y) contains supplementary material, which is available to authorized users.

## Background

Skin and soft tissue infections (SSTIs) are a preventable cause of morbidity and mortality among people who inject drugs (PWID) [1, 2]. Timely health care access for injecting-related SSTIs is suboptimal, indicating a dearth of acceptable interventions [3, 4]. A recent systematic review evidences wide variation in lifetime prevalence (6–69%) with up to a third of PWID reporting SSTIs within the past month [5]. SSTI-related complications are a leading cause of hospitalisation among PWID [4, 6–10], with hospital admission rates doubling in the USA between 1993 and 2010 [4] and increasing annually in the UK since 2012 [10]. In the UK, 36% of PWID report a recent symptom of a bacterial infection [11], with 10% admitted to hospital with SSTI complications annually [7]. Most of these hospitalisations are avoidable and are often due to delays in primary care access [7, 12, 13]. Recurrence of SSTIs among PWID is common and associated with repeat hospital visits, poor antibiotic adherence, surgical intervention, hospital discharge against medical advice and social factors such as homelessness [14, 15]. Complications associated with delayed SSTI care include sepsis, gangrene, endocarditis, chronic venous ulcers and amyloid A (AA) amyloidosis—potentiating surgical debridement, limb amputation, renal failure and death [6, 13, 16–19]. Economic implications are substantive; costs to the US health care system were approximately US$193 million in 2001 and costs to the UK health care system were assessed to be approximately £77 million per annum between July 2005 and July 2009 in 2012 [8].

Injecting-related SSTIs are a ‘hidden epidemic of suffering’ [20]. They impact the most marginalised: those who are homeless or unstably housed and those who are often economically insecure and living with multiple morbidities [21]. Women who inject drugs are often disproportionately affected [5, 11, 21]. SSTI complications are exacerbated by and can entrench experiences of social exclusion [22]. The stigma, shame, pain, unpleasant odour and mobility restrictions associated with complications, such as chronic leg ulcers, can restrict social integration, access to care and reduction of illicit drug use [3, 19, 21, 22]. Knowledge among PWID about SSTI care and complications is reportedly poor [23, 24], with practices such as lancing abscesses, inappropriate antibiotic use and poor antibiotic adherence potentially increasing wound severity, duration and recurrence [22, 25–27].

A research and policy focus on blood-borne viruses among PWID has foreclosed attention to this concern. Hepatitis C and HIV prevention is a primary remit of harm reduction services [28]. Resource constraints, due to political pressures and austerity measures in the countries such as the US and the UK, further hamper the ability of services to offer SSTI care and advice [29, 30]. Contextual information to inform interventions is crucial, given the geographical variation in injecting patterns, drug forms, service availability and associated SSTI risk [4, 31]. Published research on SSTI among PWID in the UK is primarily epidemiological, demonstrating population and health care burden [7, 12, 13, 21, 32, 33], but providing little insight into reasons for delayed care access. The scant UK qualitative literature addressing injecting-related SSTI focuses on venous ulcers [22, 34]. To date, no published studies have focused on the barriers and facilitators to timely SSTI care access among PWID in the UK.

SSTI-related complications such as AA amyloidosis are also poorly understood. AA amyloidosis results from persistent inflammation, a feature of untreated SSTIs. Evidence indicates that it has become the predominant cause of progressive renal disease among PWID [35]. If not effectively treated, AA amyloidosis can lead to renal failure, necessitating dialysis or kidney transplantation [36]. PWID experience considerable difficulties adhering to dialysis treatment, with a median survival time from diagnosis of 19 months in the UK [16]. This is a potentially preventable condition; early risk screening, regular wound care and injecting cessation can prevent and reverse disease progression [16]. Prevalence of AA amyloidosis among PWID is unknown, and no UK drug treatment services are known to currently provide routine AA amyloidosis risk screening or early intervention.

The limited extant literature indicates that there is an urgent need for research to investigate the barriers and facilitators to expedient SSTI care among PWID, including the feasibility of AA screening in drug treatment settings and referral to specialty care, to inform effective and acceptable SSTI care interventions. This paper describes the conceptual framework, methodological design and proposed analysis for the mixed-methods multi-phase study. Care and Prevent will generate an estimate of SSTI burden and AA amyloidosis prevalence among PWID using drug treatment services in London, with implications for the development of screening recommendations and SSTI interventions among PWID.

### Conceptual framework

#### Counterpublics and care

We draw on two orientating concepts: ‘counterpublic health’, attentive to the role of *corporeal learning* in sustaining *collective change* among marginalised populations [37], and an ‘ethic of care’, attentive to the *situated rationalities* informing *care practices* [38]. A ‘counterpublic health’ ethos recognises that public health goals, and the notions of health inscribed within them, do not speak to all ‘publics’. This recognition is both political and pragmatic: it works to identify and enhance the processes through which people look after themselves and, by drawing on embodied practice, promote the diffusion of collective change. Sex- and pleasure-positive HIV/AIDS interventions informed by gay community practice provide an example [37]. An ethic of care is attuned to specificities of practice in context: the environments, relationships and rationalities informing risk and protective practice. In this way, study aims are both applied and theoretical: to enable the *practical* development of health care interventions for PWID and to advance sociological theories of *care and counterpublics*. Commensurate with both aims is a focus on *practice*, with analysis attuned to the social practices and relational networks within which care is embedded as well as the alternative rationalities that can inform these practices.

#### Participatory research

A participatory research paradigm informs the study. This can be characterised by two core elements: ‘the specific quality of interaction between those conducting research and those whose lives are the focus of the research’ and ‘an inherent and often explicit connection between research and social action, the former designed to support the latter’ ([39], p. 117). Our participatory approach seeks to enable participants’ ownership and co-production of the research process [40] rather than just facilitate consultative forms of ‘involvement’ in research. Members of the affected community are active in the initiation, design and process of the study and will work with the research team to translate findings into meaningful and transformative practice. The principal investigator, coming from a PWID background [41], has an established track record of working with marginalised communities in the design, implementation and outcomes of research.

#### Positive deviance

Our focus on care and protection, as well as risk, is informed by a positive deviance methodology as operationalised in previous research by the study PI [42–46]. Most research investigating injecting-related harms focuses on ‘incident’ cases. In ‘positive deviance’ designs, the approach is the reverse, that is, to explore accounts of *protection* in contexts of high risk in order to learn about the practices shaping *avoidance* of infection and *resilience* to risk. In phase 4 of the study, we will purposively sample PWID who have comparable injecting histories but differing experience (extensive vs none/limited) of SSTIs. A dual focus on protection and risk enables investigation into successful SSTI self-care practices among PWID: crucial for informing the development of community acceptable and effective interventions. We are interested in how injecting-related wound care is cultivated and sustained in contexts of social and economic marginalisation and the conditions under which this care is constrained or becomes undone. Life history interviews and timelines will be employed and co-constructed to enable exploration of the interrelationships informing SSTI prevention, occurrence and chronicity over time. These methods, by broadening enquiry beyond the injecting history, also allow exploration of indirect social conditional factors not proximal to risk [42–45].

## Methods

Care and Prevent (‘Promoting skin and soft tissue infection care and preventing AA amyloidosis renal failure among people who inject drugs in the United Kingdom: a mixed-methods multi-phase study’) is a UK National Institute of Health Research (NIHR)-funded study seeking to improve SSTI prevention, care and treatment interventions for PWID. This includes assessing the feasibility of screening, diagnosis and treatment referral for AA amyloidosis in UK drug treatment services.

### Approach

**Fig. 1:**
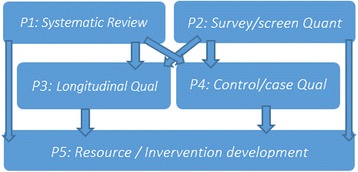
Multi-phase design

This multi-phase study employs mixed methods to address six primary objectives. We aim to (1) assess the evidence for AA amyloidosis among PWID; (2) test the feasibility of screening for AA amyloidosis in drug treatment services; (3) estimate the prevalence of SSTI and AA amyloidosis among PWID in London; (4) identify barriers and facilitators to the AA amyloidosis care pathway; (5) understand the factors informing SSTI risk, protection and care among PWID; and (6) develop SSTI and AA amyloidosis harm reduction resources in collaboration with PWID and service providers. These objectives are operationalised across the five phases. Phases 1 (assessing the evidence) and 2 (survey and screening) run concurrently. These are followed by two qualitative phases, also running concurrently: 3 (the AA amyloidosis pathway) and 4 (SSTI risk and care). Phase 5, incorporating collaborative resource development workshops, finalises the study, drawing on analyses and findings from the previous phases (see Fig. 1).

### Study phases, sites and recruitment

#### Phase 1: assessing the evidence

We will conduct a systematic review on AA amyloidosis among PWID. The details of the methods and findings of systematic review will be published elsewhere [47].

#### Phase 2: survey development

We developed an 83-item computer-assisted questionnaire, through an iterative process involving the research team, project meetings and comparisons with extant surveys, e.g. the UK unlinked anonymous survey, with the objective of assessing the prevalence of key measures and identifying modifiable behavioural risks, and risk factors, for both SSTI and AA amyloidosis among PWID participants (see Additional file 1). Question domains cover injecting and substitution treatment history; drug preparation and hygiene practices; injecting frequency and administration sites; SSTI symptoms and diagnosis; SSTI health care access and hospitalisations; other diagnosed health conditions; demographics, including homelessness history; and screening outcome, referral and consent for follow-up contact. Of note are detailed questions regarding acidifier use (type, duration and amount) for drug injection preparation, given characteristics of the brown heroin (base) predominant in the UK, and the authors (MH and DC) published hypothesis that overly acidic drug solution contributes to venous damage and SSTI risk among this population [48].

The questionnaire has been uploaded onto hand-held tablets using Open Data Kit (ODK) software. Questionnaires were pilot-tested with a small sample of PWID (*n* = 5) to check for problems with interpretation of questions, question flow, sensitive/problematic questions and completion time, and suggestions for improvement were incorporated into the final instrument. Questionnaires are researcher-administered (MH or RB), to aid consistency and reduce participant burden—particularly given the variable literacy of the study population. We have incorporated photographs of cellulitis, abscesses and venous disease for participants to refer to when indicating type and severity of current and previous SSTIs. We will take close-up, anonymous, photographs of participants’ current SSTIs with their consent. These will be uploaded into the participant’s anonymised questionnaire file, enabling comparison with participant self-identified SSTI attribution and severity. On completion, questionnaire data are transferred to the secure London School of Hygiene & Tropical Medicine (LSHTM) ODK server, protecting against loss or theft of mobile device.

### Screening and referral

Alongside the questionnaire, participants will be asked to provide a urine sample, to be screen tested for proteinuria, using an albumin to creatinine ratio measure. Point-of-care testing is conducted using a portable CLINITEX Status Analyzer machine and Microalbumin Reagent Strips. Where possible, all samples with an abnormal reading are sent for laboratory testing verification through the respective recruiting services. Participants receive CLINITEX testing results immediately; those with an albumin to creatinine ratio of > 30 mg/mmol receive a referral to the National Amyloidosis Centre (NAC) for specialist assessment. Peer support for NAC appointment attendance is provided through Groundswell, a homeless peer advocacy service (http://groundswell.org.uk/). Trained Groundswell peer advocates will contact participants prior to their first appointment, assess their needs regarding attendance, accompany them to and from the day-long appointment and provide support for duration.

The NAC assessment process is standard UK National Health Service (NHS) care [49]. Clients will be asked to consent to their assessment outcome details being shared with the research team, but no additional tests or procedures will be carried out for the study. NAC diagnostic practice involves an SAP scintigraphy (99% specificity and sensitivity for AA amyloidosis). Participants with amyloid deposition on SAP scintigraphy will be diagnosed with AA amyloidosis and receive monitoring. Those with evidence of high amyloid burden and/or advanced kidney disease will be referred for dialysis treatment. Those with low or moderate amyloidosis burden will be closely monitored and advised by NAC providers to cease injecting (supported by opiate substitution therapy referral) and resolve any current SSTIs in order to prevent disease progression. Monthly blood measurements of serum amyloid A protein (SAA) will ascertain disease progression or stabilisation. Diagnostic and treatment uptake data for each case will be collected by NAC providers and made available for analysis, with participants’ consent. Participants with positive proteinuria screen but no amyloid will be referred to a non-NAC kidney specialist.

### Sample size, eligibility criteria and recruitment strategy for phase 2

We aim to recruit 400 PWID for phase 2. The sample size has been calculated based on feasibility (numbers attending the clinics and likely response rate) and on an estimated low prevalence outcome (5% +proteinuria) but common risk factor (untreated SSTI). The sample size will be sufficient to estimate prevalence of +proteinuria ± 2% and, allowing for some attrition, to follow through the diagnostic and treatment pathway (~ 15 participants).

Phase 2 eligibility criteria are psychoactive drug injection history, client of the recruiting service, aged 18 and over, capable of providing informed consent and English language proficiency. Broad eligibility criteria (ever injecting, rather than current injecting or SSTI history) protect against bias: enabling generalisable prevalence estimates of SSTIs and AA amyloidosis risk. Recruitment sites are drug treatment services located in Central, North, South and West London. London has been chosen due to high prevalence of PWID, and varied service locations enables access to diverse PWID communities. Recruitment is facilitated through flyers on service waiting room walls and provided by service staff to eligible clients. Participants are provided with a £10 supermarket voucher for their time. Members of the research team attend each site for pre-determined periods each week and made themselves available to answer client questions, assess eligibility and conduct data generation.

#### Phase 3: the AA amyloidosis pathway

We will qualitatively explore experiences of the AA amyloidosis care pathway. Phase 3 participants will be sampled from recruitment site clients previously diagnosed with AA amyloidosis (*n* = 5–10) and those referred to the NAC, from phase 2, who consent to qualitative follow-up (*n* = ~ 10). Previously diagnosed clients will be invited to participate in an in-depth interview about their experiences of diagnosis, dialysis treatment and protective and risk practices prior to and following diagnosis. Participants from phase 2 will be interviewed at baseline (prior to assessment), at assessment and 3–4 months after assessment. Longitudinal data collection allows prospective exploration of the barriers and facilitators to diagnostic and treatment care, including wound resolution, dialysis and injecting cessation support needs. A topic guide orientates around these issues, with a focus on experiences of the diagnostic and treatment pathway. This process also allows observation of attrition. With consent, participants will be accompanied by the lead researcher and the Groundswell peer to the day-long diagnostic appointment. Non-participant observations will be recorded through field notes. Observations will explore the dynamics of patient-provider and patient-peer communication processes in situ, also enabling triangulation through observing events which are recounted in interviews.

#### Phase 4: SSTI protection and risk

We will concurrently employ a ‘positive deviance’ methodology to explore practices and contexts informing SSTI protection and risk. A comparative sample of age and injecting history matched PWID, with and without SSTI, will be recruited from phase 2 survey sample (*n* = 30). Snowball sampling will enable additional recruitment of participants (*n* = 15–20) not in touch with drug services. Participants will be interviewed once, with the option for follow-up (up to 60 interviews among 45–50 participants). Qualitative interviews will explore practices and knowledge of SSTI risk and care, the conditions which facilitate or undermine SSTI care, barriers and facilitators to timely treatment access, and experiences of health care systems. It is important that this sample is distinct from, and not dependent on, the phase 3 sample. This allows a focus specifically on SSTI and protects against uncertainties of the phase 3 sample size.

### Sample size eligibility criteria and recruitment for phases 3 and 4

Phase 2 participants who consent to follow-up contact will be stratified to enable purposive sampling for the qualitative study phases (3 and 4). Phase 3 sample size is dependent on proteinuria testing results; preliminary calculations estimate ~ 12 participants. For phase 4, we will purposively sample for variation in gender, age, housing situation, injecting duration, drugs injected, SSTI and hospitalisation history. Participants not in touch with drug treatment services will be recruited through snowball sampling. Women who inject drugs are often disproportionately affected by SSTI, and face additional barriers to service access, and therefore will be actively recruited. The flexibility in phase 4 sample size (*n* = ~ 50) allows both theoretical sampling and saturation [50].

#### Phase 5: resource development

Phase 5 draws on the existing evidence base (phase 1) and empirical data (phases 2–4) to inform the co-construction of SSTI and AA amyloidosis intervention resources and recommendations with stakeholders and members of the affected community. A diagrammatic logic model will describe anticipated resource delivery mechanisms, components, mechanisms of impact and intended outcomes. This will be informed by and act as a guide to collaborative decision-making on resource content and design. Workshops with PWID and providers at each site will be held at each phase of output design. Budget is provided and collaboration developed (film-maker, designer) to enable production of hard copy booklets and videos both for PWID and providers with dissemination enabled through social media platforms, drug services, treatment provider and drug user networks.

### Mixed-methods analysis

Data generated for each phase will be analysed separately. While single-phase analysis does not preclude each phase building and drawing on previous analysis, it provides a modest safeguard against dependence on previous phase completion and validity [51]. Data triangulation, or mixed-methods interpretation [51], will occur after phase 3 and 4 (qualitative study) completion; drawing inferences from both quantitative and qualitative findings in relation to the overarching research question, triangulation of mixed-methods data will prioritise complementarity (findings greater than the sum of their parts), while also attentive to convergence and dissonance [52].

Phase 2 analysis of quantitative survey and clinical data will initially be descriptive, with the aim of generating overall prevalence estimates and according to participant characteristics (demographics, injecting history and risk practices, SSTI history). Cross-sectional analyses will be carried out using logistic regression and will examine associations with two main outcomes: positive screen for proteinuria and SSTI occurrence and severity. If the prevalence of AA amyloidosis is high enough, this will also be an outcome in our models. Predictors include participant characteristics and risk behaviours. With the outcome of +proteinuria, frequency of SSTI is the main predictor to be tested.

Analysis of phase 3 and 4 qualitative data will follow grounded theory principles [7], with verbatim transcripts coded as collected in order to inform the direction of subsequent interviews, coding, case selection, memo and theory generation. Grounded theory is a systematic and comparative method for studying processes [8], particularly useful for generating substantive theory (grounded in data) [8] with transferable potential for other sites and conditions. Coding will be implemented in a step-wise process; comprising open coding, focused coding, category mapping and theme development. Through this process, we will move from the particular to the general, the localised to the abstract, with a focus on developing conceptual density [53] and transferable mid-range theory.

### Ethics and governance

Ethical approvals have been received from the NHS Health Research Authority [17/LO/0872], the London Bridge Research Ethics Committee [17/LO/0872] and the LSHTM Observational Research Ethics Committee [12021]. Informed written consent will be obtained from each participant prior to enrolment and after information and an explanation of the study purpose and procedures is given. Participants will be asked to consent to the following: (1) provide a urine sample to be tested for protein and results shared with research team and abnormal results being shared with their general practitioner (GP); (2) to complete a confidential questionnaire about their injecting practices and health history; (3) to provide a confidential photo of their skin infection if applicable; (4) to the anonymisation of data used in study reports, papers and publications; (5) to the secure storage of anonymised data in a data repository for use by other researchers; (6) if referred to the NAC, the results of any assessments to be shared with the research team; (7) to be contacted for follow-up interview if necessary and to be sent study outputs if desired; (8) and if contacted for an in-depth-interview, for it to be recorded and the transcript securely stored and anonymised extracts be used in reports, papers or publications. The research is being carried out in accordance with the principles, policies, procedures and guidelines contained in the LSHTM Good Research Practice policy.

## Results and discussion

The Care and Prevent study is innovative; it is the first to assess screening feasibility and the prevalence of positive proteinuria (as a marker for AA amyloidosis) among PWID accessing drug treatment services. This has potentially significant implications. AA amyloidosis is an under-recognised SSTI complication for which early intervention is available but not employed [16, 47]. The current evidence base for AA amyloidosis among PWID spans back over four decades, yet epidemiological data are weak; case reports predominate [47]. No UK data exists on the prevalence of AA amyloidosis among PWID, and no international published research prospectively explores early intervention for AA amyloidosis or barriers to current care pathways. In the UK context where AA amyloidosis risk is not screened for, most PWID are referred to specialist assessment after renal failure is discovered. Dialysis adherence is poor [16, 54], and deaths from sepsis common [16]. Screening may enable early intervention and avert renal failure. This has significant cost savings and patient benefit potential.

### Current status of the study and amendments

We commenced phase 2 data generation in September 2017. An unexpectedly high proportion (40/100) of participants recruited up to 22 November 2017 have had test results indicating microalbuminuria (albumin: creatinine ratio 3.4–33.9 mg/mmol). This necessitated a study amendment for the development of a GP referral letter. Although microalbuminuria is not an a priori outcome measure (we are looking for macroalbuminuria > 30 mg/mmol), it indicates raised risk for cardiovascular or progressive kidney disease, and, therefore, with participants consent, we are notifying their GP. In order to unpack the significance of this finding, if microalbuminuria prevalence persists, we have also added questions pertaining to smoking and inhalation history (of tobacco and other drugs), family history of kidney disease, and occurrence of scabies (associated with microalbuminuria in indigenous populations [55]) and blood in the urine. This was felt necessary given, general population prevalence of microalbuminuria ranging from 3.3% (women in the UK) [56] to 7.2% (the Netherlands) [57] and, in the USA, 6% (men) and 9.7% (women) [58]. While hepatitis C is associated with albuminuria and renal insufficiency, this does not appear to account for preliminary findings—with prevalence of microalbuminuria in people with hepatitis C reported at 11–12% [59].

### Study implications

Our study has dual, inter-related aims. The findings will determine whether AA amyloidosis risk screening is warranted and envisage that screening recommendations, if required, will be for targeted implementation rather than among all PWID at drug treatment services. The linkage of individual questionnaire and screening data facilitates the analysis of risk associations for specific screening recommendations. Our study also provides insight into the nature and extent of the health harms experienced by PWID in drug services, with a specific focus on SSTI prevention and care. Regardless of AA amyloidosis risk outcome, we know that timely health care access for SSTIs is suboptimal among PWID in the UK and places a huge burden not only on the health care system but also on individual PWID in terms of suffering, stigma and loss of mobility.

The value of our methodology is that we seek not only to understand risk among those with SSTI, but also on how protection is enabled. Participants include those with lengthy injecting durations who have managed to ‘stay safe’ while living on the street; their involvement and insight is invaluable for the development of community-acceptable interventions.

Our output content and format will be determined by study findings and community acceptability. Resources and recommendations are to be developed, drawing on the evidence from phases 1 to 4, in collaboration with PWID service user representatives and study participants. We envisage that intervention scope will encompass not only SSTI and AA amyloidosis educational and awareness resources, but also practical interventions to enable self-care, such as wound care kits, and to support injecting route transitions. Review evidence indicates that favourable outcomes for AA amyloidosis among PWID require not only effective SSTI treatment but also injecting cessation [47]. It is unclear, however, whether it is the act of injecting per se that exacerbates inflammation (and poor AA amyloidosis outcomes) or the injecting risk environment which potentiates harm. Phase 3, where we will follow those diagnosed with AA amyloidosis, may shed light on this question and provide insight into the unique barriers faced by PWID in maintaining the burden of dialysis care.

### Strengths and limitations

Our study is, by necessity, exploratory. The prevalence of AA amyloidosis is unknown among PWID in the UK; formative data are required before implementation of any larger scale screening study. As such, the sample size for phase 2 is small (*n* = 400) but calculated as large enough to provide sufficient power for our outcome measures. Study sites are all situated in London, and the sample population reflects those who engage with drug treatment services, limiting generalisability of findings to other locations or countries. PWID who do not seek advice/treatment from drug treatment services may have different experiences with SSTI than those who do engage. Some recall bias is to be expected, as those who experience, since those who experience more severe SSTI may remember it differently from those who do not. Some underreporting of stigmatised practices and health conditions is also to be expected. Qualitative data, however, may generate transferable insight, particularly regarding barriers to SSTI care and interventions to facilitate protection and care among marginalised and homeless populations.

## Conclusions

We report the protocol for, and implementation status of, the NIHR-funded study ‘Care and Prevent’. This study innovates through implementing AA amyloidosis risk screening among PWID in drug treatment services, following those diagnosed through the care pathway and employing a positive deviance methodology to explore SSTI risk, protection and care among PWID. Given the preponderance of published literature and interventions targeting blood-borne viruses among PWID, there is an urgent need for innovative research and interventions for SSTI among PWID—conditions which cause considerable suffering and result in a high uptake of ambulatory care. AA amyloidosis is a devastating yet preventable sequelae of SSTI among PWID; we aim to ascertain if risk screening will benefit, or unnecessarily burden, drug service clients.

## Additional file

Additional file 1: Variables measured in the Care and Prevent questionnaire. (DOCX 16 kb)

## Abbreviations

LSHTM: London School of Hygiene & Tropical Medicine
NAC: National Amyloidosis Centre
NHS: National Health Service
ODK: Open Data Kit
PWID: People who inject drugs
