# Supplementary material for: ‘Care and Prevent’: rationale for investigating skin and soft tissue infections and AA amyloidosis among people who inject drugs in London
Source: Harm Reduct J. 2018 May 8;15:23. doi: 10.1186/s12954-018-0233-y (PMC5941602; doi:10.1186/s12954-018-0233-y)
Supplement: Supplementary file 1 — Variables measured in the Care and Prevent questionnaire. (DOCX 16 kb) [file 12954_2018_233_MOESM1_ESM.docx]

**Additional file 1 Variables measured in the Care and Prevent Questionnaire**

| **Domains** | **Variable/measure** |
| --- | --- |
| Demographics | Age;  gender;  ethnicity;  country of birth |
| Socio-economic status | Main source of income;  Main accommodation arrangement;  History and duration of homelessness |
| Injecting and treatment history | Age first injected;  Injecting cessation history;  Opiate substitution therapy status/duration |
| Types of drugs injected, preparation, and injecting body site and injecting frequency | Recent (12 month) injecting drug use including main drug injected;  Duration injecting main drug;  Main type of filter used;  History and main use of acids;  Quantity of vitamin C and citric acid used to prepare a £10 bag of heroin;  Injecting frequency per week;  Body site injected (ever, recent-12 months, and main site);  Main method of injecting;  Usual needle insert times required |
| Hygiene practices | Reuse of filters;  Handwashing before injecting;  Use of alcohol swabs on injection site before injecting;  Reuse of needles or syringes;  Cleaning of needles or syringes before reuse;  Substance used to clean needles or syringes |
| SSTI history, frequency, duration, body site | Cellulitis/abscesses/ulcers/venous disease history, duration, frequency;  Main body site infected with SSTI |
| SSTI health care access practices and hospitalisation | Action taken when first noticed SSTI;  Source of medical help for SSTI;  Ever hospitalized for SSTI;  Antibiotic prescription received;  Source of antibiotics;  Completion of prescribed course;  Required regular wound dressing;  Frequency of wound dressing attendance |
| Diagnosis/history of health conditions and health behaviour | Diagnosis of various health conditions;  Smoking of tobacco and other drugs;  History of scabies, kidney disease, blood in urine |
